# Controlling excimer formation in indolo[3,2,1-*jk*]carbazole/9*H*-carbazole based host materials for RGB PhOLEDs†
†Electronic supplementary information (ESI) available: NMR spectra; S_0_ → S_1_ NTO; DSC, TGA, and CV data; PL and EL spectra. CCDC 1568697. For ESI and crystallographic data in CIF or other electronic format see DOI: 10.1039/c8tc03537g


**DOI:** 10.1039/c8tc03537g

**Published:** 2018-08-31

**Authors:** Chenyang Zhao, Thomas Schwartz, Berthold Stöger, Fraser J. White, Jiangshan Chen, Dongge Ma, Johannes Fröhlich, Paul Kautny

**Affiliations:** a State Key Laboratory of Polymer Physics and Chemistry, Changchun Institute of Applied Chemistry, Chinese Academy of Sciences , Changchun , 130022 , China; b University of Science and Technology of China , Hefei , 230026 , China; c Institute of Applied Synthetic Chemistry, TU Wien , Getreidemarkt 9/163 , A-1060 Vienna , Austria . Email: paul.kautny@tuwien.ac.at; d X-Ray Centre, TU Wien , Getreidemarkt 9 , A-1060 Vienna , Austria; e Rigaku Oxford Diffraction , Unit B6 Chaucer Business Park, Watery Lane, Kemsing , Sevenoaks , UK; f Institute of Polymer Optoelectronic Materials and Devices, State Key Laboratory of Luminescent Materials and Devices, South China University of Technology , Guangzhou , 510640 , China . Email: msjschen@scut.edu.cn

## Abstract

Three novel materials (**5CzICz**, **Cz_2_ICz** and **Cz_3_ICz**), based on the indolo[3,2,1-*jk*]carbazole and 9*H*-carbazole building blocks, with high triplet energies (*E*_T_ > 2.80 eV) and good thermal stability (*T*_g_ > 101 °C) were synthesized, characterized and applied as host materials in PhOLED devices. In course of the preparation of the materials, an improved protocol for the synthesis of the indolo[3,2,1-*jk*]carbazole moiety has been developed. The careful molecular design of the title compounds allowed to avoid excimer formation of the indolo[3,2,1-*jk*]carbazole subunits in thin films. Therefore, the improved molecular design broadened the applicability of indolo[3,2,1-*jk*]carbazole host materials and significantly enhanced the efficiency of PhOLED devices based on these derivatives compared to previously reported indolo[3,2,1-*jk*]carbazole based compounds. Accordingly, employing the newly developed materials red (CE_max_: 32.7 cd A^–1^, PE_max_: 31.0 lm W^–1^, EQE_max_: 20.4%), green (CE_max_: 81.0 cd A^–1^, PE_max_: 87.4 lm W^–1^, EQE_max_: 21.5%) and blue (CE_max_: 35.5 cd A^–1^, PE_max_: 39.9 lm W^–1^, EQE_max_: 18.0%) PhOLED devices with a remarkably low efficiency roll-off at 1000 cd m^–2^ (**Cz_2_ICz** – red: 5%; green: 0%; blue: 6%) were fabricated.

## Abstract 2

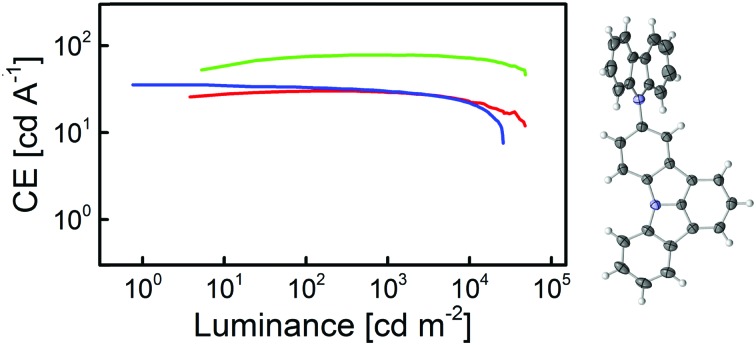
Excimer formation in indolo[3,2,1-*jk*]carbazole based host materials is controlled by molecular design and the developed materials are employed in efficient red, green and blue PhOLED devices.

## Introduction

Since the first reports on electroluminescence,^1^ the technology of Organic Light Emitting Diodes (OLEDs) has been steadily developed.^2^–^6^ In particular, the introduction of heavy transition metal complexes, which are capable of efficient phosphorescence, boosted the efficiency of OLED devices.^7^,^8^ These metal complexes can harvest singlet and triplet excitons simultaneously in phosphorescent OLEDs (PhOLEDs) and thus significantly enhance the internal quantum efficiency compared to traditional purely fluorescent organic materials.^9^,^10^ In 2012 Adachi *et al.* presented a new class of highly efficient emitters based on the concept of thermally activated delayed fluorescence (TADF).^11^ For this purpose, purely organic donor–acceptor materials with a carefully controlled, small energy splitting between the excited singlet and excited triplet state have been developed. The small energy difference between the two excited states allows for the promotion of the triplet excitons to singlet excitons at room temperature by means of thermal energy. Thus, TADF emitters can match the efficiency of phosphors without the incorporation of heavy metals.^12^,^13^

Both, phosphorescent and TADF emitters, need to be widely dispersed in a host material to prevent concentration quenching, particularly at a high brightness. Moreover, these host materials provide an enhanced thermal stability and improved charge transport properties of the emitting layer. The triplet energy (*E*_T_) of the host material has to be higher than the *E*_T_ of the emitting material, to confine the excited states on the emitter.^14^–^16^

The 9*H*-carbazole building block has been widely employed in the design of host materials owing to favorable hole transport properties.^17^–^23^ In particular, 4,4′-bis(*N*-carbazolyl)-1,1′-biphenyl (CBP) has been frequently employed as host material for various phosphorescent emitters.^14^ However, CBP features two major drawbacks: (i) a low *E*_T_ of 2.56 eV,^24^ which prohibits the application of CBP as host material for blue emitters and (ii) a low glass transition temperature (*T*_g_) of 62 °C.^25^

**Scheme 1:**
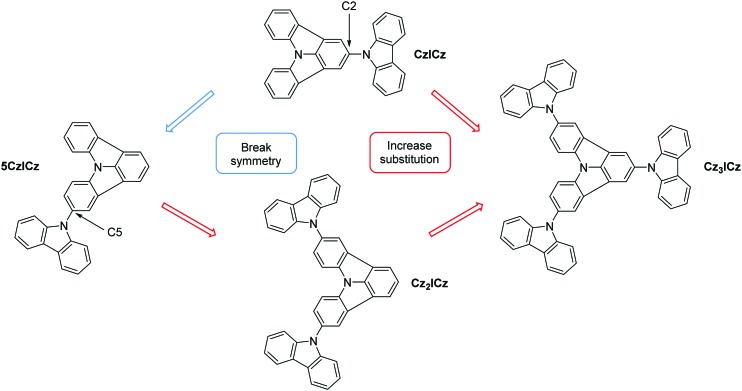
Design concept of the novel ICz based host materials.

Recently, we introduced indolo[3,2,1-*jk*]carbazole (ICz) as a novel, fully planarized arylamine building block to the field of organic electronics.^26^,^27^ The efficient preparation of the ICz motive employing a C–H activation strategy^28^ enables the application of this structural motive in functional organic materials.^26^,^29^ The incorporation of the ICz scaffold into the CBP backbone yielded C2 substituted ICz based host materials (Scheme 1) with high *E*_T_s and good thermal stability.^30^

Furthermore, the ICz motive has been employed as weakly electron accepting unit in the design of efficient blue thermally activated delayed fluorescent (TADF) emitters.^31^ Substitution of ICz with a cyano group increased the electron accepting properties of the building block and allowed for the preparation of deep blue TADF emitters.^32^

However, the planar structure of ICz allows for a pronounced, intermolecular interaction between individual host molecules. Consequently, the formation of excimers in thin films of the developed host materials was observed, which prohibited the efficient application of the majority of the developed compounds in blue PhOLEDs.^30^ The formation of excimers in 9*H*-carbazole containing materials has been previously described.^33^–^38^ However, excimer formation was also observed in ICz based host materials without 9*H*-carbazole substituents.^30^ Noteworthy, the energy of the observed excimers was approximately 2.25 eV and thus lower than the energy of the reported 9*H*-carbazole based excimers (2.4–2.6 eV).^34^,^35^,^37^,^38^ Hence, it is plausible to assume that the nature of these excimers is different from those found in 4,4′-bis(*N*-carbazolyl)-1,1′-biphenyl (CDBP) and related materials.^36^,^38^

Excimers significantly impact the efficiency of blue and, to an even larger extent, deep blue emitters and thus also the practical applicability of OLED devices. Therefore, it is of crucial importance to understand the mechanism behind excimer formation in order to control this undesired interaction. Consequently, the aim of our current work is to improve the molecular design of the ICz based host materials and investigate the effect of the molecular modifications on the excimer formation. The effect of molecular design on the excimer formation in thin films is of particular interest and a deeper understanding of the structural features that lead to excimer formation is crucial to control and avoid this undesired effect. Therefore, compound **CzICz** (Scheme 1) was revisited, as it exhibited the best performance among the previously reported ICz materials. Notably, the excimers observed for ICz-based host materials were located on the ICz moiety as discussed above. Accordingly, an improved molecular design has to focus on the prevention of excimer formation on this particular molecular building block. The tendency for excimer formation depends strongly on the intermolecular interaction between the individual molecules and their respective alignment.^39^ Thus, the design concept of the new ICz based materials relies on the prevention of intermolecular interactions of the individual ICz moieties (Scheme 1). To hinder the ordered arrangement of the molecules, we intended to decrease the symmetry of **CzICz** by altering the substitution position of the attached 9*H*-carbazole motive from the 2-position to the 5-position of the ICz moiety, thus removing the twofold rotation axis (**5CzICz**). Furthermore, we introduced additional 9*H*-carbazole groups in order to increase the steric demand of the substituents and shield the ICz from adjacent molecules (**Cz_2_ICz**, **Cz_3_ICz**). Consequently, the ICz is continuously moved from the periphery of the molecule to its center.

## Results and discussion

### Synthesis

**Scheme 2:**
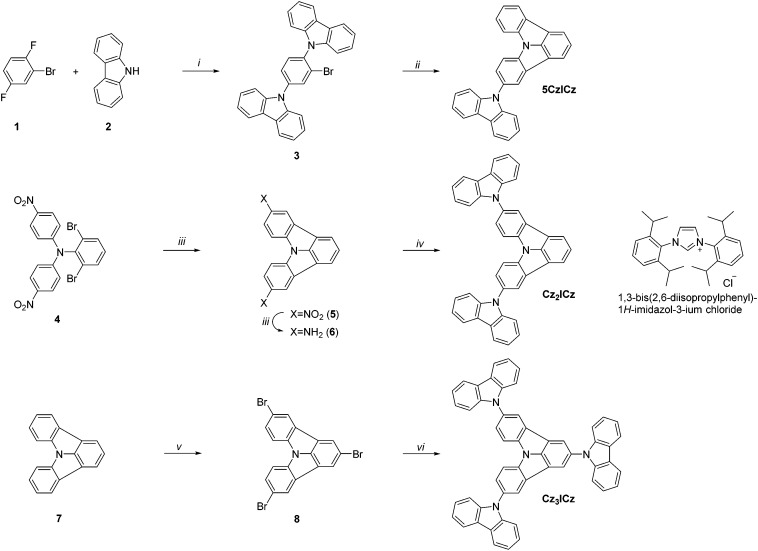
Synthetic approach towards the ICz based host materials: (i) Cs_2_CO_3_, DMSO, 120 °C; (ii) Pd(OAc)_2_, ligand·HCl, K_2_CO_3_, DMA, 130 °C; (iii) SnCl_2_·2H_2_O, DMSO/H_2_O (10 : 1), 80 °C; (iv) NaO*t*Bu, 2-bromo-2′-iodo-1,1′-biphenyl, Pd_2_(dba)_3_, dppf, toluene, reflux; (v) Br_2_, DCM (dichloromethane); (vi) 9*H*-carbazole, CuSO_4_·5H_2_O, K_2_CO_3_, 250 °C.

Three different approaches were pursued in the preparation of the target materials, owing to the varying substitution positions of the ICz moiety (Scheme 2). Alternative strategies had to be developed to realize the desired substitution patterns, as nucleophilic substitution preferentially occurs in the 2-position of ICz. In the case of **5CzICz**, both amine groups were simultaneously introduced by nucleophilic substitution of **1** with 9*H*-carbazole. Subsequently, C–H activation was utilized to establish the ICz moiety by ring closure. Notably, a novel procedure was employed to accomplish the ring closure. Previously, a preformed Pd-catalyst with an N-heterocyclic carbene ligand^40^,^41^ was employed. In the novel protocol, the precursor salt of the ligand (1,3-bis(2,6-diisopropylphenyl)-1*H*-imidazol-3-ium chloride; Scheme 2) was directly applied in combination with Pd(OAc)_2_ forming the catalyst *in situ*. Following this procedure, **5CzICz** was obtained in a good yield of 79% after column chromatography. The new procedure is preferred compared to the previously described methodology because it renders the separate formation of the catalyst unnecessary while retaining the high yields of the ring closing step. In analogy, a nucleophilic substitution of 1-bromo-4-fluorobenzene with 2,6-dibromobenzenamine was the first step in the preparation of **Cz_2_ICz**. Subsequently, twofold C–H activation established the ICz scaffold. Notably, dinitro-ICz (**5**) was obtained in an excellent yield of 93%, highlighting the versatility of the novel methodology for the ring closure. Using SnCl_2_·2H_2_O, **5** was reduced to diamine **6**. Following the Nozaki approach, the two 9*H*-carbazole moieties were formed by Pd catalyzed Buchwald–Hartwig amination, yielding **Cz_2_ICz**. The preparation of **Cz_3_ICz** was accomplished by threefold bromination of ICz followed by the introduction of the 9*H*-carbazoles in an Ullmann reaction. All target materials were characterized by ^1^H and ^13^C NMR as well as high resolution mass spectrometry.

**Fig. 1:**
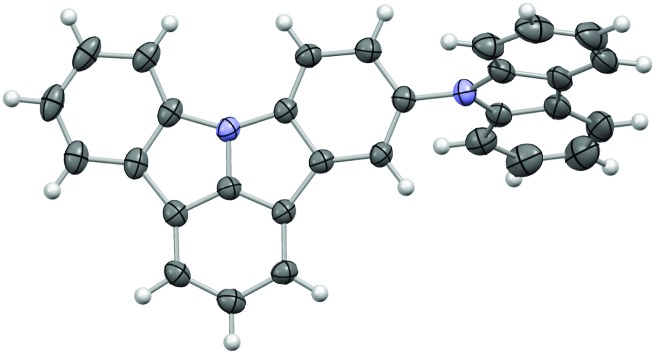
Molecular structure of **5CzICz**; C and N atoms are represented by grey and blue ellipsoids drawn at 50% probability level, H atoms by white spheres of arbitrary radius.

In the case of **5CzICz**, the molecular structure was determined by single crystal analysis (Fig. 1). The ICz and 9*H*-carbazole moieties are virtually flat [maximum distance to least squares (LS) planes defined by the non-H atoms: 0.043(2) Å (ICz) and 0.029(4) Å (9*H*-carbazole)] and distinctly inclined to each other [angle between LS planes: 66.01(6)°]. Notably, the substitution pattern of the ICz influences the torsion angle between the two planar subunits. Whereas the torsion angle in C2 substituted **CzICz** (Scheme 1) was 54.07(5)°,^30^ it is increased in C5 substituted **5CzICz**.

The thermal properties of the developed host materials were investigated by differential scanning calorimetry (DSC, Fig. S16, ESI†) and thermogravimetric analysis (TGA, Fig. S17, ESI†). The *T*_g_ of the materials increased with progressive 9*H*-carbazole substitution from **5CzICz** (101 °C) to **Cz_2_ICz** (171 °C) and **Cz_3_ICz** (327 °C). The *T*_g_ of all compounds is significantly higher compared to CBP,^25^ which can be attributed to the increased rigidity of the ICz scaffold and benefits the morphological stability of thin films of the materials. Furthermore, all materials exhibited an exceptional thermal stability with decomposition temperatures (*T*_d_: corresponding to 5% mass loss during thermogravimetric analysis) of 450 °C or higher.

The energies of the HOMO and LUMO levels of the newly developed ICz based host materials were determined by cyclic voltammetry (CV, Fig. S18–S20, ESI†). All compounds exhibited irreversible oxidation, as typically found for 9*H*-carbazole and ICz derivatives, due to the instability of the formed oxidation products.^30^,^42^ The energy of the HOMO levels span a narrow range between –5.57 and –5.63 eV, comparable to **CzICz**.^30^ The LUMO levels of **5CzICz** and **Cz_2_ICz** are located at –2.47 eV, while the LUMO of **Cz_3_ICz** is located slightly lower at –2.59 eV. These HOMO and LUMO energy levels indicate no significant injection barriers for holes and electrons from adjacent layers in OLED devices.

### Theoretical calculations

**Fig. 2:**
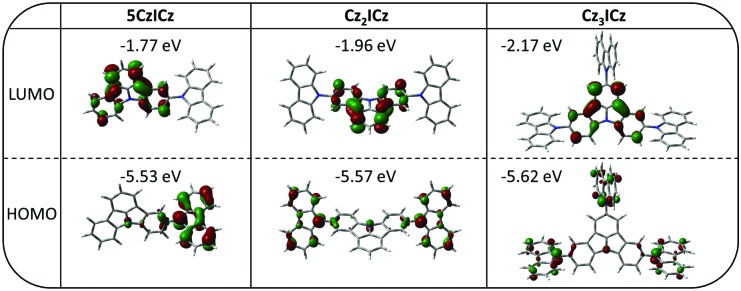
Spatial distribution of the HOMOs and LUMOs of the new host materials.

To obtain a more detailed understanding of the molecular electronics, we employed density functional theory (DFT). The calculated energy of HOMO levels are in good agreement with the experimentally determined values with a maximum deviation of 0.04 eV (Table 1). In contrast to the HOMO levels the energy of LUMO levels are systematically overestimated. Furthermore, the theoretical calculations predict a continuous decrease of the LUMO energy from **5CzICz** to **Cz_2_ICz** and **Cz_3_ICz**, which is only observed to a small degree for **Cz_3_ICz** in the experimental data. A clear separation of the frontier molecular orbitals is observed for all three materials (Fig. 2). The HOMOs are mainly located on the 9*H*-carbazole units with some contribution from the central nitrogen of the ICz scaffold. This behavior is in line with the stronger electron donating nature of the 9*H*-carbazole compared to ICz.^26^ Likewise, the LUMO levels of all materials are exclusively located on the ICz moieties.

**Table 1:** Physical data of the synthesized host materials

	*T*_g_/*T*_rc_/*T*_m_/*T*_d_^*a*^ [°C]	Opt. BG^*b*^ ^,^^*c*^ [eV]	*λ*_max,PL_ ^*c*^ [nm]	HOMO/LUMO [eV]		*E*_T_ ^*f*^ [eV]
				Exp.^*d*^	Cal.^*e*^	
**5CzICz**	101/181/244/450	3.19	410	–5.57/–2.47	–5.53/–1.77	2.83
**Cz_2_ICz**	171/n.o.^*g*^/308/>500	3.08	420	–5.54/–2.47	–5.57/–1.96	2.82
**Cz_3_ICz**	327/n.o.^*g*^/n.o.^*g*^/>500	3.02	432	–5.63/–2.59	–5.62/–2.17	2.80

^*a*^Determined by thermogravimetric analysis and differential scanning calorimetry; *T*_g_: glass transition temperature; *T*_rc_: recrystallization temperature; *T*_m_: melting point; *T*_d_: decomposition temperature corresponding to 5% mass loss.

^*b*^Optical bandgap determined from the absorption onset.

^*c*^Measured in DCM solutions (5 μM) at room temperature.

^*d*^Calculated from the onset of the oxidation and reduction peak observed during cyclic voltammetry.

^*e*^Calculated applying density functional theory level (B3LYP/6-311G**).

^*f*^Determined from the highest vibronic transition in solid solutions of a mixture of DCM/toluene/MeOH (10/10/1) at 77 K.

^*g*^Not observed.

**Fig. 3:**
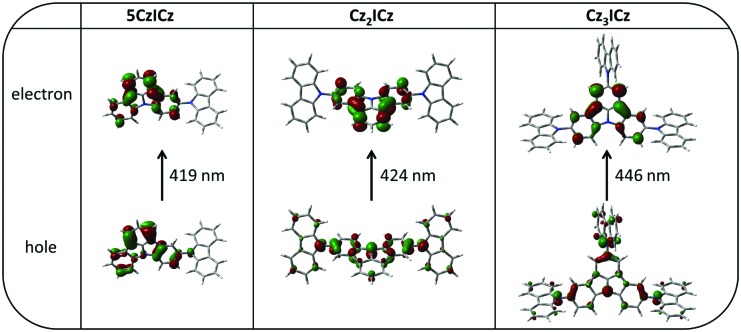
Natural transition orbitals (NTOs) of the S_0_ → T_1_ transition calculated by TD-DFT at the optimized S_0_ geometries.

To learn more about the nature of the excited states, we used time dependent density functional theory (TD-DFT) to calculate the S_0_ → S_1_ and S_0_ → T_1_ transitions. The natural transition orbitals (NTOs) of the first excited singlet state of all three materials closely resemble the HOMOs and LUMOs of the compounds (Fig. S15, ESI†). Accordingly, the excited holes are localized on the 9*H*-carbazole units, whereas the excited electrons are localized on the ICz motive. Therefore, the S_1_ is an intramolecular charge transfer (ICT) state. In contrast, for the S_0_ → T_1_ transition the excited hole and the excited electron are both localized on the ICz moiety with a small contribution to the excited holes coming from the nitrogen atoms of the 9*H*-carbazole units and to some extent also from the 9*H*-carbazoles in the case of **Cz_3_ICz** (Fig. 3). Thus, the T_1_ state can be considered a mainly localized excited (LE) state centered on the ICz with a light charge transfer character in the case of **Cz_2_ICz** and **Cz_3_ICz**.

### Photophysical properties

**Fig. 4:**
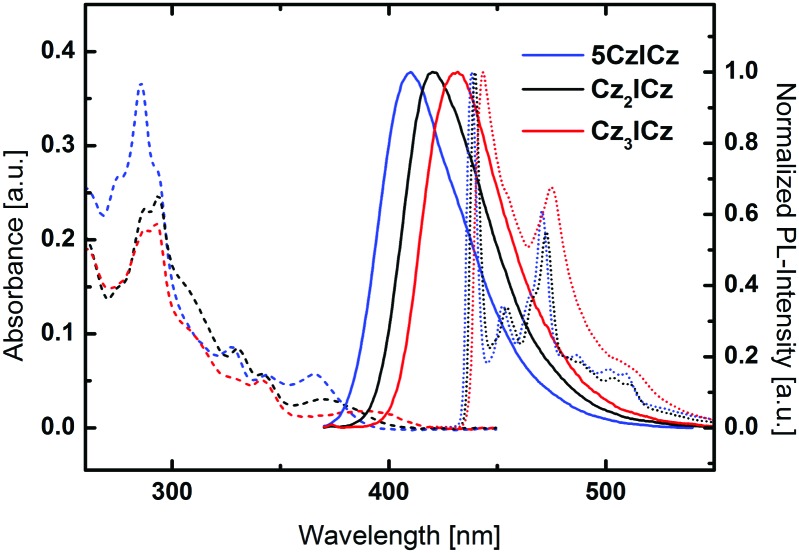
UV/vis absorption (dashed lines), normalized fluorescence (solid lines) and normalized phosphorescence (dotted lines) spectra of the ICz based host materials. UV/vis absorption and normalized fluorescence spectra were recorded in DCM solutions (5 μM) at room temperature. Phosphorescence spectra were recorded in solid solutions of DCM/toluene/MeOH (10/10/1) at 77 K. F = fluorescence; P = phosphorescence.

UV/vis absorption, room temperature photoluminescence and low temperature phosphorescence spectra were recorded to investigate the photophysical properties of the newly developed materials (Fig. 4). All three materials featured two intense absorption bands between 286 and 297 nm, which can be attributed to π–π* transitions centered on the 9*H*-carbazole and ICz moieties, respectively. Moreover, two transitions of the conjugated backbone of the molecules are observed between 327 and 330 nm and between 341 and 344 nm as small peaks or shoulders for all three molecules. Notably, the absorption wavelength of the lowest energy transition is continuously red-shifted from **5CzICz** (365 nm) to **Cz_2_ICz** (369 nm) and **Cz_3_ICz** (389 nm), owing to the increased size of the π-conjugated system. Accordingly, the absorption onsets are shifted towards lower energy from 3.19 (**5CzICz**) to 3.08 (**Cz_2_ICz**) and 2.97 eV (**Cz_3_ICz**). Compared to CBP, the optical bandgaps are significantly reduced, which is expected to decrease the driving voltage and thus yield high power efficiencies in PhOLED devices.^30^

All materials exhibited unstructured fluorescent emission in DCM (dichloromethane) at room temperature. This observation is in good agreement with the theoretical calculations, which suggest an ICT S_1_ state. Furthermore, the absorption and emission properties of the materials in solvents with different polarity were investigated (Fig. S24–S26, ESI†). While the absorption was basically independent of the solvent polarity, all materials exhibited a distinct solvatochromism and the emission maxima were shifted towards higher wavelengths with increasing solvent polarity. This behavior indicates a polar excited state and thus confirms the assumption of an ICT S_1_ state.

In analogy to the absorption onsets, the wavelengths of the fluorescence maxima are red-shifted from 410 (**5CzICz**) to 420 (**Cz_2_ICz**) and 432 nm (**Cz_3_ICz**). In contrast to the slightly different fluorescence, **5CzICz** and **Cz_2_ICz** featured nearly the same vibronically resolved phosphorescent emission. The maxima of the highest energy vibronic transitions are located at 438 and 440 nm, corresponding to *E*_T_s of 2.83 and 2.82 eV, respectively. The presence of a vibronically resolved emission confirms the LE nature of the T_1_ state, as suggested by the theoretical calculations. The phosphorescence spectrum of **Cz_3_ICz** appears less structured compared to the other two materials, due to the increased CT character. Nonetheless, the same transitions can be found with a slight overall red-shift leading to an *E*_T_ of 2.80 eV. From these findings it can be concluded that the nature of the excited triplet state of the three materials is indeed basically the same and localized on the ICz moiety as predicted by the theoretical calculations.

Notably, the *E*_T_s of the compounds are significantly increased compared to CBP^24^ and in the same range as those of previously reported ICz derivatives.^30^ Therefore, the developed materials are potential host materials for blue phosphorescent emitters.

**Fig. 5:**
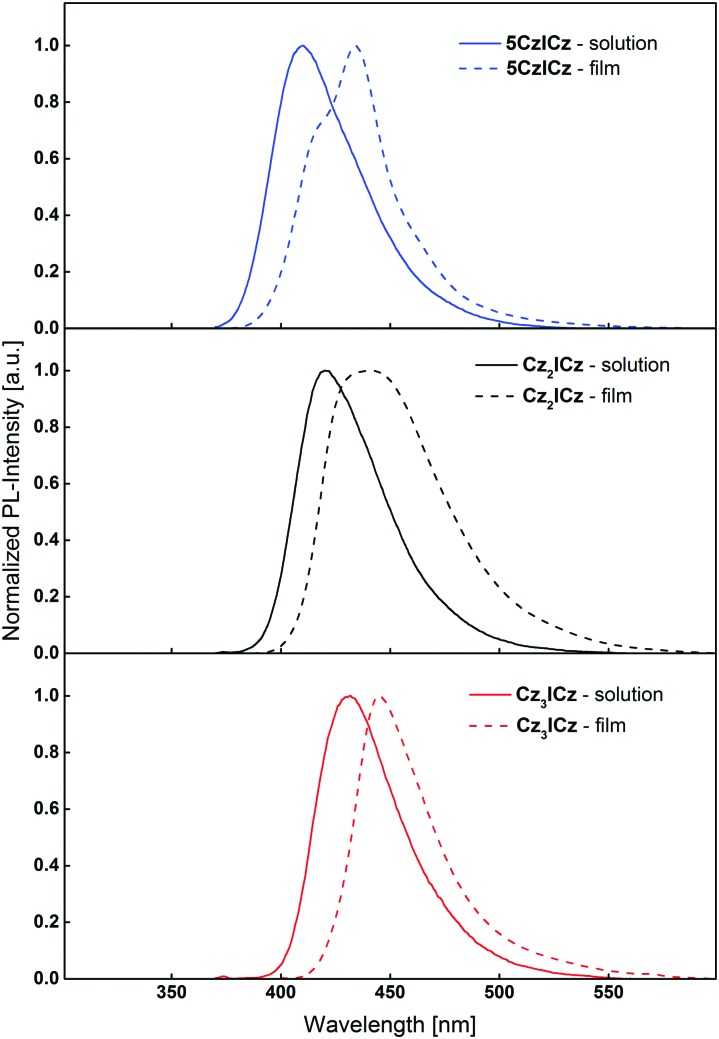
Normalized fluorescence and spectra of diluted DCM solutions and thin films of the novel ICz based host materials.

To understand the effects of the molecular modifications on excimer formation, the photoluminescence of thin films of the newly designed ICz based host materials were recorded. Compared to the emission from diluted DCM solutions, the film fluorescence of the compounds is slightly red-shifted (Fig. 5), owing to the altered chemical environment of the individual molecules. **Cz_3_ICz** exhibited a rather sharp emission band. This behaviour may be attributed to the fact that the excited state is most shielded by the threefold carbazole substitution (Fig. S15, ESI†) of **Cz_3_ICz** and thus less influenced by the chemical environment. Most notably, there are no additional low energy emission bands in the emission of the thin films, as previously observed for the C2 substituted ICz based host materials.^30^ The absence of this long wavelength emission, which is a sign for the formation of excimers,^34^,^36^ proves that the presented design approach of the novel materials indeed sufficiently suppresses the intermolecular interaction of the individual ICz units and therefore prevents the formation of excimer states in thin films.

### Electroluminescent properties

**Fig. 6:**
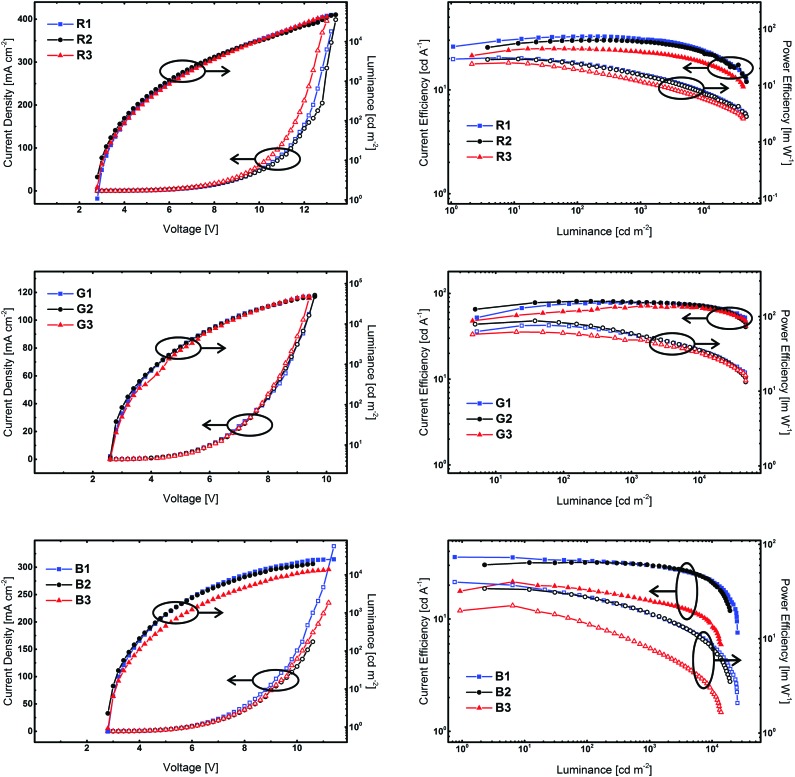
Key electroluminescent properties of the prototype devices employing **5CzICz**, **Cz_2_ICz**, and **Cz_3_ICz** as host materials. Left: Current density–voltage–luminance (current density: hollow symbols; luminance: full symbols), right: current efficiency–luminance–power efficiency (current efficiency: full symbols; power efficiency: hollow symbols).

Finally, the developed compounds were investigated regarding their universal applicability as host materials in red, green, and blue PhOLEDs. Accordingly, employing **5CzICz** (**1**), **Cz_2_ICz** (**2**), and **Cz_3_ICz** (**3**) as host materials red (**R**), green (**G**), and blue (**B**) PhOLED prototype devices with the following architecture have been fabricated: (i) **R**: ITO/MoO_3_ (8 nm)/TAPC (50 nm)/EML (host material, Ir(MDQ)_2_(acac) (5%), 12 nm)/BPhen (70 nm)/LiF/Al; (ii) **G**: ITO/MoO_3_ (8 nm)/TAPC (60 nm)/EML (host material, Ir(ppy)_2_(acac) (8%), 12 nm)/BPhen (70 nm)/LiF/Al; (iii) **B**: ITO/MoO_3_ (8 nm)/TAPC (50 nm)/EML (host material, FIrpic (10%), 15 nm)/BmPyPB (45 nm)/LiF/Al. TAPC was utilized as a hole transporting layer and BPhen was employed as an electron transporting and hole blocking layer. In the case of blue devices, BPhen was replaced by BmPyPB, owing to the *E*_T_ of BmPYPB (2.69 eV)^43^ being higher compared to the *E*_T_ of BPhen (2.56 eV).^44^ Current density–voltage–luminance and current efficiency–luminance–power efficiency curves are depicted in Fig. 6. The key electroluminescent properties of all devices are summarized in Table 2. The observed emission exclusively originated from the employed phosphorescent emitter and representative emission spectra of the PhOLED devices are given in the ESI.†

**Table 2:** Key electroluminescent properties of the synthesized host materials

	*V*_on_ [V]	CE^*a*^ [cd A^–1^]	PE^*a*^ [lm W^–1^]	EQE^*a*^ [%]
**R1**	2.8	30.7/27.0/32.7	15.8/10.1/31.0	18.7/16.1/20.2
**R2**	2.8	29.0/25.9/30.0	15.4/9.7/29.3	19.3/17.0/20.4
**R3**	2.8	22.9/20.4/24.9	11.5/7.4/25.2	16.3/14.2/17.6
**G1**	2.6	77.7/75.1/78.0	57.1/42.1/76.3	20.7/20.1/20.8
**G2**	2.6	79.3/75.6/81.0	59.1/42.5/87.4	21.0/20.1/21.5
**G3**	2.6	69.2/69.7/71.8	48.5/38.8/62.2	18.6/18.6/19.2
**B1**	2.8	29.8/25.1/35.5	18.3/11.5/39.9	15.0/12.7/18.0
**B2**	2.8	30.1/25.8/31.7	18.5/11.7/34.0	14.6/12.5/15.4
**B3**	2.8	14.6/11.5/21.4	7.9/4.4/22.5	7.0/5.5/10.2

^*a*^Measured at a brightness of 1000 cd m^–2^/measured at a brightness of 5000 cd m^–2^/maximum efficiency.

Notably, all devices featured a very low onset voltage (*V*_on_) of 2.6 V (**G1–3**) or 2.8 V (**R1–3** and **B1–3**). The *V*_on_ is thus slightly lower than the electrochemically determined HOMO–LUMO gap and indicates efficient charge injection into the emitting layer. The slightly higher *V*_on_ of the red devices compared to the green devices is attributed to direct charge trapping on the red emitter.

Red devices **R1–3** featured a good performance with a maximum external quantum efficiency (EQE_max_) of up to 20.4% for **R2**. Among these devices, **R1** employing **5CzICz** as host material featured the highest maximum current efficiency (CE_max_) of 32.7 cd A^–1^ and the highest maximum power efficiency (PE_max_) of 31.0 lm W^–1^. Notably, the CE of **R1** decreased only slightly with increasing brightness and the CEs at 1000 and 5000 cd m^–2^ were 30.7 and 27.0 cd A^–1^, corresponding to an efficiency roll-off of 6% and 17%, respectively. A good efficiency at high brightness is of particular importance for practical applications and thus is a relevant characteristic of newly developed host materials. The CE_max_ and PE_max_ of **R2** and **R3** of 30.0 and 24.9 cd A^–1^ and 29.3 and 25.2 lm W^–1^ were slightly lower compared to the CE_max_ and PE_max_ of **R1**. The efficiency roll-off of **R2** and **R3**, however, was similar compared to **R1**.

In analogy to **R1–3**, green devices **G1–3** exhibited good performance with very high CE_max_ of 81.0 cd A^–1^ for **G2**, 78.0 cd A^–1^ for **G1**, and 71.8 cd A^–1^ for **G3**, corresponding to EQE_max_ of 21.5%, 20.8%, and 19.2%. Owing to the low driving voltages, the devices also displayed high PE_max_ of 76.3 (**G1**), 87.4 (**G2**), and 62.2 lm W^–1^ (**G3**). Most strikingly however, **G1** showed hardly any efficiency roll-off at 1000 cd m^–2^. It still exhibited an excellent CE of 75.1 cd A^–1^ and a PE of 42.1 lm W^–1^ at 5000 cd m^–2^ corresponding to an EQE of 20.1% and an efficiency roll-off of 4%. Likewise, the efficiency roll-off in **G2** was low. At 5000 cd m^–2^**G2** displayed a CE of 75.6 cd A^–1^ and an EQE of 20.1% and thus an efficiency roll-off of 7%. In the case of device **G3**, the situation is slightly different as the efficiency of **G3** increased at lower and intermediate brightness and its CE_max_ was reached at approximately 1700 cd m^–2^, before the CE slowly decreased. As can be seen in Fig. 6 (right column, middle row), the CE and PE of **G3** are slightly lower compared to **G1** and **G2** at lower brightness but approach those of the other two devices at higher brightness. Therefore, G**3** exhibited the lowest efficiency roll-off of only 3% at 5000 cd m^–2^ among all green devices.

Notably, the performance of the green devices employing the newly developed host materials could be significantly improved compared to our previous results with C2 substituted ICz derivatives.^30^ While the EQEs were increased from 15 to 20% the PE of the devices employing the new materials were doubled over the whole brightness range highlighting the effect of the improved molecular design of the title compounds. The high CE and EQE of the red and green devices indicate efficient exciton confinement on the employed phosphorescent emitters. Moreover, the high power efficiencies result from the low bandgap of the materials and efficient charge injection into the emitting layer. These device characteristics are vital for the practical applications and underline the importance of the ICz based architecture as a novel platform for the development of efficient host materials.

While the three developed materials featured comparable results for red and green emitters, the situation is different for blue emitting FIrpic. Device **B1** employing **5CzICz** exhibited a very satisfying CE_max_ of 35.5 cd A^–1^ corresponding to an EQE_max_ of 18.0% and a high PE_max_ of 39.9 lm W^–1^, while the CE_max_ (31.7 cd A^–1^) and EQE_max_ (15.4%) of **B2** were somewhat lower. At higher brightness, **B1** and **B2** featured similar efficiency values. On the other hand, the performance of **Cz_3_ICz** in **B3** was considerably lower with an EQE_max_ of 10.2%. Moreover, **B3** displayed the highest efficiency roll-off among the investigated materials. In summary, the observed efficiencies of devices **B1** and **B2** are very satisfying. In analogy to the green devices, the novel design concept proved superior to the C2 substitution pattern. Not only were all new materials applicable as host materials for FIrpic, but also **B1** and **B2** exhibited increased device performance compared to our previous results.^30^

## Conclusions

In this study, we report on three novel indolo[3,2,1-*jk*]carbazole based host materials for PhOLEDs. Our systematic investigations regarding the substitution position of the indolo[3,2,1-*jk*]carbazole moiety revealed that the formation of excimers on the indolo[3,2,1-*jk*]carbazole subunits in thin films of the materials can be prevented by a careful molecular design. Clearly, the suggested design strategy for indolo[3,2,1-*jk*]carbazole based host materials starting with C5 substitution proved to be superior compared to C2 substitution. Accordingly, the efficiency of PhOLED devices based on the developed materials could be improved compared to previously reported derivatives and all new materials were applicable as host materials in blue devices. Furthermore, the efficiency roll-off of the fabricated prototype devices was remarkably low. Therefore, we are convinced that these investigation will guide the design of new indolo[3,2,1-*jk*]carbazole derivatives for applications as host materials and beyond.

## Experimental section

### General information

All reagents and solvents were obtained commercially and used without further purification. Anhydrous solvents were prepared by filtration through drying columns. The water content of purchased DMA was determined by Karl Fischer titration and corrected to 1000 ppm for C–H activation reactions. Column chromatography was performed using silica 60 (Merck, 40–63 μm). NMR spectra were recorded on a Bruker DRX-600 MHz Spectrometer. Absorption and photoluminescence measurements were conducted using a Thermo Scientific NanoDrop One^C^ Microvolume UV/vis spectrophotometer and a PerkinElmer LS 55 fluorescence spectrometer, respectively. Solution measurements were recorded employing DCM solutions (5 μM). Phosphorescence spectra were recorded at 77 K using solid solutions of the materials in a mixture of DCM/toluene/MeOH (10/10/1) with a delay of 0.1 ms or thin films of the compounds and a delay of 1 ms. Thermal analysis was carried out with a heating rate of 10 K min^–1^. Differential scanning calorimetry (DSC) was performed using a Netzsch DSC 200 F3 Maia and a Netzsch simultaneous thermal analyzer (STA 449 F1 Jupiter) was employed for thermogravimetric analysis (TGA), working with pierced aluminum pans and under N_2_ (TGA) or argon (DSC) atmosphere. Cyclic voltammetry (CV) was done using a three electrode configuration consisting of a Pt working electrode, a Pt counter electrode, and an Ag/AgCl reference electrode and a PGSTAT128N potentiostat provided by Metrohm Autolab B.V. Measurements were carried out in a 0.5 mM solution in anhydrous DCM (oxidation) or ACN (reduction) employing Bu_4_NBF_4_ (0.1 M) as supporting electrolyte. Prior to the measurements, the solutions were purged with nitrogen for approximately 15 minutes. The HOMO and LUMO energy levels were calculated from the onset of the oxidation and reduction peaks, respectively. The onset potential was determined by the intersection of two tangents drawn at the background and the rising of the oxidation and reduction peaks. High resolution mass spectroscopy was carried out using an Agilent 1100/1200 HPLC in combination with an Agilent 5230 AJS ESI-TOF mass spectrometer.

### Synthetic details

**1**, **2**, and 9*H*-carbazole have been purchased from commercial suppliers and used without further purification. Indolo[3,2,1-*jk*]carbazole^26^ and 2-bromo-2′-iodo-1,1′-biphenyl^45^ have been prepared according to published procedures.

#### 9-(2-Bromo-4-9*H*-carbazol-9-ylphenyl)-9*H*-carbazole (**3**)

**1** (4.83 g, 25.0 mmol, 1.0 eq.), **2** (8.36 g, 50.0 mmol, 2.0 eq.), and Cs_2_CO_3_ (19.55 g, 60.0 mmol, 2.4 eq.) were suspended in 25 ml of DMSO in a three necked flask and heated to 120 °C for 48 h (TLC, no further conversion). Since no full conversion was observed after 48 h, additional Cs_2_CO_3_ (1.63 g, 5.0 mmol, 0.2 eq.) was added. After a total reaction time of 72 h, the reaction mixture was cooled to room temperature, poured on 200 ml of water and the precipitated crude product was filtered. **3** (5.58 g, 11.4 mmol, 46%) was obtained as a colorless solid after column chromatography (light petrol : DCM 85 : 15 → 83 : 17). ^1^H-NMR (600 MHz, CD_2_Cl_2_): *δ* = 8.22–8.20 (m, 4H), 8.16 (d, *J* = 2.3 Hz, 1H), 7.83 (dd, *J* = 8.3, 2.3 Hz, 1H), 7.77 (d, *J* = 8.2 Hz, 1H), 7.65 (d, *J* = 8.2 Hz, 2H), 7.53–7.48 (m, 4H), 7.39–7.34 (m, 4H), 7.26 (d, *J* = 8.2 Hz, 2H) ppm. ^13^C NMR (150 MHz, CD_2_Cl_2_): *δ* = 141.4, 140.9, 139.7, 135.9, 132.7, 132.7, 127.8, 126.9, 126.7, 125.1, 124.3, 123.8, 121.2, 121.0, 120.9, 120.8, 110.6, 110.2 ppm. HRMS (ESI): *m*/*z* calculated for C_30_H_19_BrN_2_: 486.0732 [M]^+^; found: 486.0723 [M]^+^.

#### 5-(9*H*-Carbazol-9-yl)indolo[3,2,1-*jk*]carbazole (**5CzICz**)

**3** (3.41 g, 7.0 mmol, 1.0 eq.) and K_2_CO_3_ (1.94 g, 14.0 mmol, 2.0 eq.) were suspended in degassed DMA (30 ml, 1000 ppm H_2_O) in a three necked flask under argon atmosphere. Subsequently, 1,3-bis(2,6-diisopropylphenyl)-1*H*-imidazol-3-ium chloride (60 mg, 0.14 mmol, 2 mol%) and Pd(OAc)_2_ (31 mg, 0.14 mmol, 2 mol%) were added under argon counterflow. The reaction mixture was heated to 130 °C until full conversion (TLC, 24 h), cooled to room temperature and partitioned between H_2_O and DCM. The black residue was removed by filtration and the aqueous layer was repeatedly extracted with DCM. The combined organic layers were dried over anhydrous Na_2_SO_4_ and concentrated under reduced pressure. **5CzICz** (2.25 g, 5.5 mmol, 79%) was obtained as a colorless solid after column chromatography (light petrol : DCM 85 : 15). Single crystals of **5CzICz** were obtained after crystallization from toluene. ^1^H-NMR (600 MHz, CDCl_3_): *δ* = 8.31 (d, *J* = 1.9 Hz, 1H), 8.22–8.19 (m, 3H), 8.12–8.11 (m, 2H), 8.04 (d, *J* = 7.4 Hz, 1H), 7.99 (d, *J* = 7.9 Hz, 1H), 7.74 (dd, *J* = 8.3, 2.0 Hz, 1H), 7.64–7.61 (m, 2H), 7.47–7.42 (m, 5H), 7.35–7.32 (m, 2H) ppm. ^13^C NMR (150 MHz, CDCl_3_): *δ* = 144.4, 141.6, 138.6, 137.6, 131.5, 131.3, 130.0, 127.0, 126.0, 125.9, 123.3, 123.2, 122.4, 122.1, 120.3, 120.1, 119.8, 119.8, 118.8, 118.0, 112.9, 112.2, 109.7 ppm (one carbon atom was not detected). HRMS (ESI): *m*/*z* calculated for C_30_H_18_Br_2_: 406.1470 [M]^+^; found: 406.1468 [M]^+^.

#### 2,6-Dibromo-*N*,*N*-bis(4-nitrophenyl)benzenamine (**4**)

The preparation of **4** was carried out as a modification of a published procedure.^46^ 2,6-Dibromobenzenamine (3.67 g, 14.6 mmol, 1.0 eq.), 1-bromo-4-fluorobenzene (4.66 g, 33.0 mmol, 2.3 eq.), and Cs_2_CO_3_ (10.75 g, 33.0 mmol, 2.3 eq.) were suspended in 75 ml of DMSO and heated to 130 °C overnight. After cooling to room temperature, the reaction mixture was poured on 300 ml of H_2_O and the precipitated crude product was filtered. **4** (5.64 g, 11.4 mmol, 78%) was obtained as yellow solid after being refluxed in EtOH and subsequent filtration. Physical data according to literature.^46^

#### 5,11-Dinitroindolo[3,2,1-*jk*]carbazole (**5**)

**4** (12.33 g, 25.0 mmol, 1.0 eq.) and K_2_CO_3_ (13.82 g, 100.0 mmol, 4.000 eq.) were suspended in degassed DMA (100 ml, 1000 ppm H_2_O) in a three necked flask under argon atmosphere. Subsequently, 1,3-bis(2,6-diisopropylphenyl)-1*H*-imidazol-3-ium chloride (213 mg, 0.50 mmol, 0.02 eq.) and Pd(OAc)_2_ (112 mg, 0.50 mmol, 0.02 eq.) were added under argon counterflow. The reaction mixture was heated to 130 °C until full conversion (TLC, 48 h) and cooled to room temperature. The precipitated product was filtered and dried under reduced pressure yielding **5** (7.68 g, 23.2 mmol, 93%) as greenish solid. ^1^H-NMR (600 MHz, DMSO-d_6_): *δ* = 9.36 (d, *J* = 2.3 Hz, 2H), 8.67 (d, *J* = 8.9 Hz, 2H), 8.57 (dd, *J* = 8.9, 2.3 Hz, 2H), 8.51 (d, *J* = 7.4 Hz, 2H), 7.82 (t, *J* = 7.4 Hz, 1H) ppm. ^13^C NMR (150 MHz, DMSO-d_6_): *δ* = 145.5, 143.3, 140.8, 130.1, 125.2, 123.1, 122.8, 119.9, 117.9, 113.8 ppm.

#### Indolo[3,2,1-*jk*]carbazole-5,11-diamine (**6**)

**5** (1.32 g, 4.0 mmol, 1.0 eq.) and SnCl_2_·2H_2_O (9.04 g, 40.1 mmol, 10.1 eq.) were suspended in 22 ml of DMSO/H_2_O (10 : 1) in a three necked flask under argon atmosphere and heated to 80 °C overnight. After being cooled to room temperature, the reaction mixture was basified with 2 M NaOH and repeatedly extracted with CHCl_3_. The combined organic layers were dried over anhydrous Na_2_SO_4_ and concentrated under reduced pressure. Column chromatography (light petrol : EA 1 : 3 → EA) yielded **6** (0.64 g, 2.4 mmol, 59%) as a yellow-green solid. ^1^H-NMR (600 MHz, CD_2_Cl_2_): *δ* = 7.96 (d, *J* = 7.3 Hz, 2H), 7.65 (d, *J* = 8.5 Hz, 2H), 7.49 (t, *J* = 7.4 Hz, 1H), 7.47 (d, *J* = 2.3 Hz, 2H), 6.93 (dd, *J* = 8.5, 2.3 Hz, 2H), 3.78 (s, 4H) ppm. ^13^C NMR (150 MHz, CD_2_Cl_2_): *δ* = 145.0, 142.0, 133.1, 131.0, 122.2, 119.7, 118.8, 115.2, 112.6, 109.7 ppm. HRMS (ESI): *m*/*z* calculated for C_18_H_13_N_3_: 271.1109 [M]^+^; found: 271.1109 [M]^+^.

#### 5,11-Di(9*H*-carbazol-9-yl)indolo[3,2,1-*jk*]carbazole (**Cz_2_ICz**)

The introduction of the 9*H*-carbazole units in **Cz_2_ICz** followed a methodology based on Buchwald–Hartwig amination, which was introduced by Nozaki and co-workers.^47^**6** (0.76 g, 2.8 mmol, 1.0 eq.), 2-bromo-2′-iodo-1,1′-biphenyl (2.21 g, 6.2 mmol, 2.2 eq.), Pd_2_(dba)_3_ (513 mg, 0.56 mmol, 20 mol%), 1,1′-bis(diphenylphosphino)ferrocen (621 mg, 1.12 mmol, 40 mol%), and NaO*t*Bu (2.15 g, 22.4 mmol, 8.0 eq.) were suspended in degassed anhydrous toluene in a three necked flask under argon atmosphere and heated to reflux. After full conversion (TLC, 18 h), the reaction mixture was poured on water and repeatedly extracted with DCM. The combined organic layers were dried over anhydrous Na_2_SO_4_ and concentrated under reduced pressure. **Cz_2_ICz** (1.25 g, 2.2 mmol, 78%) was obtained as a bright yellow solid after column chromatography (light petrol : DCM 85 : 15 → 33 : 67). ^1^H-NMR (600 MHz, CDCl_3_): *δ* = 8.37 (d, *J* = 1.9 Hz, 2H), 8.22 (d, *J* = 7.8 Hz, 4H), 8.20 (d, *J* = 8.3 Hz, 2H), 8.12 (d, *J* = 7.4 Hz, 2H), 7.80 (dd, *J* = 8.3, 2.1 Hz, 2H), 7.67 (t, *J* = 7.4 Hz, 1H), 7.49–7.45 (m, 8H), 7.36–7.33 (m, 4H) ppm. ^13^C NMR (150 MHz, CDCl_3_): *δ* = 145.1, 141.5, 137.6, 131.9, 131.4, 126.3, 126.0, 123.6, 123.3, 122.6, 120.5, 120.4, 119.9, 118.4, 113.0, 109.7 ppm. HRMS (ESI): *m*/*z* calculated for C_42_H_25_N_3_: 571.2048 [M]^+^; found: 571.2053 [M]^+^.

#### 2,5,11-Tribromoindolo[3,2,1-*jk*]carbazole (**8**)

Indolo[3,2,1-*jk*]carbazole (0.48 g, 2.0 mmol, 1.0 eq.) was dissolved in 10 ml of DCM in a three necked flask and cooled to 0 °C. Br_2_ (1.05 g, 6.6 mmol, 3.3 eq.) was separately dissolved in 10 ml of DCM, cooled and then added to the solution immediately forming a precipitate. After 7 h, the precipitate was filtered and washed with DCM and aqueous Na_2_SO_3_ solution. After being refluxed in toluene and subsequent filtration, **8** (0.7 g, 1.5 mmol, 73%) was obtained as beige solid. ^1^H-NMR (600 MHz, DMSO-d_6_): *δ* = 8.59 (d, *J* = 2.0 Hz, 2H), 8.51 (s, 2H), 8.32 (d, *J* = 8.58 Hz, 2H), 7.82 (dd, *J* = 8.6, 2.0 Hz, 2H) ppm. ^13^C NMR (150 MHz, DMSO-d_6_): *δ* = 141.9, 137.1, 130.5, 130.2, 126.8, 124.1, 118.5, 115.7, 114.9 ppm (one carbon atom was not detected).

#### 2,5,11-Tri(9*H*-carbazol-9-yl)indolo[3,2,1-*jk*]carbazole (**Cz_3_ICz**)

The synthesis of **Cz_3_ICz** was accomplished analogously to a published procedure.^48^ A reaction vial was charged with thoroughly mixed **8** (1.91 g, 4.0 mmol, 1.0 eq.), 9*H*-carbazole (3.01 g, 18.0 mmol, 4.5 eq.), CuSO_4_·5H_2_O (0.15 g, 0.6 mmol, 15 mol%), and K_2_CO_3_ (2.49 g, 18.0 mmol, 4.5 eq.) and heated in a heating block to 250 °C for 72 h. After being cooled to room temperature, the solidified mixture was dissolved in DCM and H_2_O and repeatedly extracted with DCM. The combined organic layers were dried over anhydrous Na_2_SO_4_ and concentrated under reduced pressure. Most of the excess 9*H*-carbazole was removed from the crude product by vacuum sublimation. Column chromatography (light petrol : DCM 70 : 30) yielded **Cz_3_ICz** (2.25 g, 3.1 mmol, 76%) as beige solid. ^1^H-NMR (600 MHz, CDCl_3_): *δ* = 8.34 (d, *J* = 2.0 Hz, 2H), 8.24–8.23 (m, 4H), 8.21–8.19 (m, 6H), 7.86 (dd, *J* = 8.5, 2.1 Hz, 2H), 7.50–7.45 (m, 8H), 7.43–7.41 (m, 2H), 7.38 (d, *J* = 8.5 Hz, 2H), 7.35–7.30 (m, 6H). ^13^C NMR (150 MHz, CDCl_3_): *δ* = 144.0, 142.2, 141.4, 138.1, 133.6, 132.4, 131.1, 127.0, 126.0, 126.0, 123.3, 123.1, 122.9, 120.9, 120.4, 120.3, 120.0, 119.9, 119.1, 113.3, 109.6, 109.6 ppm. HRMS (ESI): *m*/*z* calculated for C_54_H_32_N_4_: 736.2627 [M]^+^; found: 736.2624 [M]^+^.

### Computational details

(TD-)DFT calculations were performed using the Gaussian 09 package^49^ applying the Becke three-parameter hybrid functional with Lee–Yang–Perdew correlation (B3LYP)^50^,^51^ in combination with Pople basis sets 6-311G(d,p) or 6-311+G(d,p).^52^ Geometry optimizations were performed in the gas phase and without symmetry constraints applying the 6-311G(d,p) basis sets. Singlet and triplet excitations were calculated using the S_0_ optimized geometry applying the 6-311+G(d,p) basis sets. Orbital plots were generated using GaussView.^53^

### Single crystal diffraction

Intensity data of **5CzICz** were collected at 100 K in a dry stream of nitrogen using Cu Kα radiation generated by microfocus tube on a Rigaku Oxford Diffraction XtaLAB Synergy diffractometer equipped with a Pilatus 200 K detector. Data reduction and absorption correction were performed with the CrysAlis^Pro^ software suite.^54^ The structure was solved with the dual-space method implemented in SHELXT^55^ and refined against *F*^2^ with SHELXL.^56^ H atoms were placed at calculated positions and refined as riding on the parent C atom.

### Device fabrication and measurement

Prior to device fabrication, the spectroscopically pure host materials were purified by crystallization from toluene (**5CzICz**, **Cz_2_ICz**, **Cz_3_ICz**) followed by sublimation (**5CzICz**, **Cz_2_ICz**). The sheet resistance of the patterned ITO-coated glass substrates used was 10 Ω per square. Before vacuum evaporation, they were completely cleaned in the ultrasonic bath using detergents and deionized water in turn. After that, the substrates were put into a vacuum drying oven at a temperature of 120 °C for 15 min. Then, they were treated with UV generated ozone for 15 min. All the organic layers were grown by thermal evaporation under high vacuum of ∼10^–4^ Pa. The evaporation rate of organic layers was 1–2 Å s^–1^ Current–voltage–brightness characteristics and EL spectra were carried out by a Keithley source measurement unit (Keithley 2400 and Keithley 2000) with a calibrated silicon photodiode and Spectrascan PR650 spectrophotometer, respectively. EQEs were obtained from the luminance, current density, and the EL spectra.

## Conflicts of interest

There are no conflicts to declare.

## Supplementary Material

Supplementary information

Crystal structure data
