# Supplementary material for: Controlling excimer formation in indolo[3,2,1-jk]carbazole/9H-carbazole based host materials for RGB PhOLEDs
Source: J Mater Chem C Mater. 2018 Aug 31;6(37):9914–24. doi: 10.1039/c8tc03537g (PMC6167949; doi:10.1039/c8tc03537g)
Supplement: Supplementary file 1 [file TC-006-C8TC03537G-s001.pdf]

Electronic Supplementary Material (ESI) for Journal of Materials Chemistry C.
This journal is © The Royal Society of Chemistry 2018

Supporting Information

Controlling Excimer Formation in Indolo[3,2,1-*jk*]carbazole/9*H*-carbazole based host materials for RGB PhOLEDs

Chenyang Zhao[§,†,$] Thomas Schwartz[§,‡] Berthold Stöger,[‖] Fraser J. White,[+] Jiangshan Chen,[†,~,*] Dongge Ma,[†,~] Johannes Fröhlich,[‡] Paul Kautny[‡,*]

*E-mail: paul.kautny@tuwien.ac.at

*E-mail:msjschen@scut.edu.cn

[†]State Key Laboratory of Polymer Physics and Chemistry, Changchun Institute of Applied Chemistry, Chinese Academy of Sciences, Changchun, 130022, China

[$]University of Science and Technology of China, Hefei, Anhui 230026, China

[‡]Institute of Applied Synthetic Chemistry, TU Wien, Getreidemarkt 9/163, A-1060 Vienna, Austria

[‖]X-Ray Centre, TU Wien, Getreidemarkt 9, A-1060 Vienna, Austria

[+]Rigaku Oxford Diffraction, Unit B6 Chaucer Business Park, Watery Lane, Kemsing, Sevenoaks, UK.

~Institute of Polymer Optoelectronic Materials and Devices, State Key Laboratory of Luminescent Materials and Devices, South China University of Technology, Guangzhou, 510640, China

[§]contributed equally to this work

1. NMR spectra

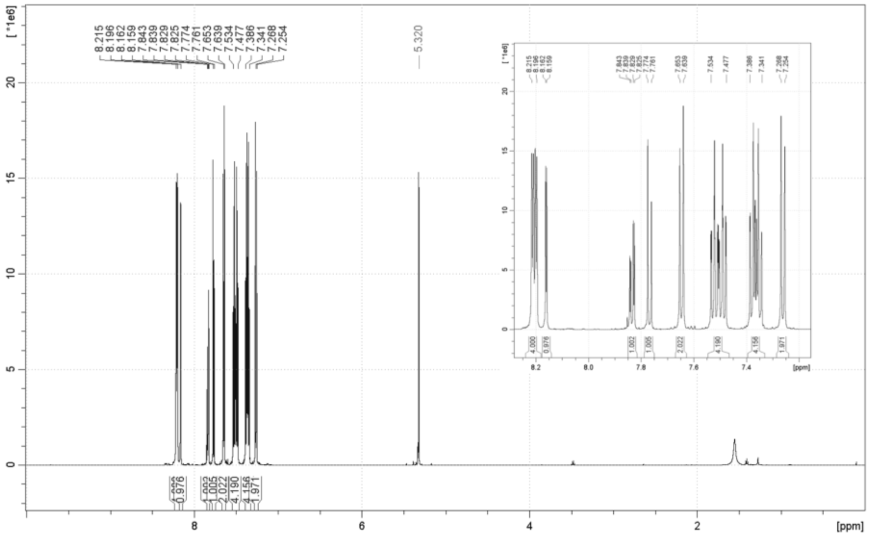

Figure S1 Proton NMR spectrum of **3**.

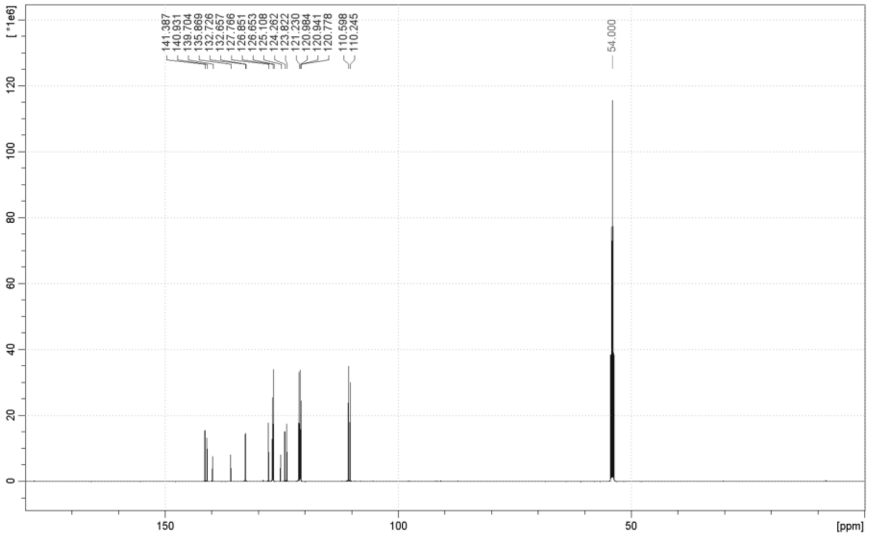

Figure S2 Carbon NMR spectrum of **3**.

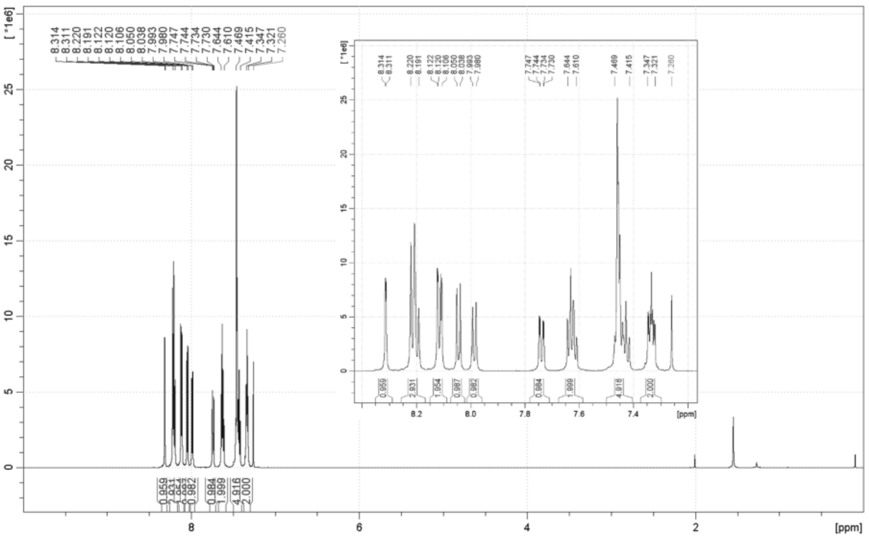

Figure S3 Proton NMR spectrum of **5CzICz**.

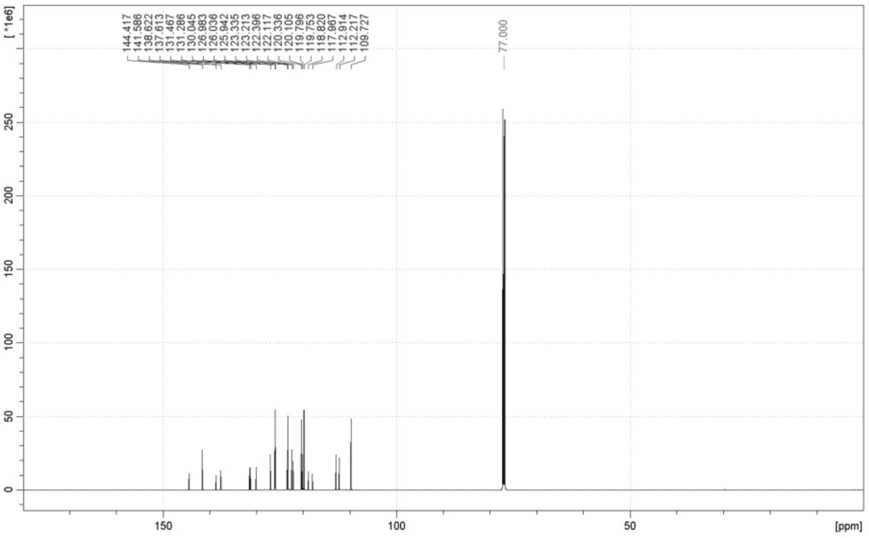

Figure S4 Carbon NMR spectrum of **5CzICz**.

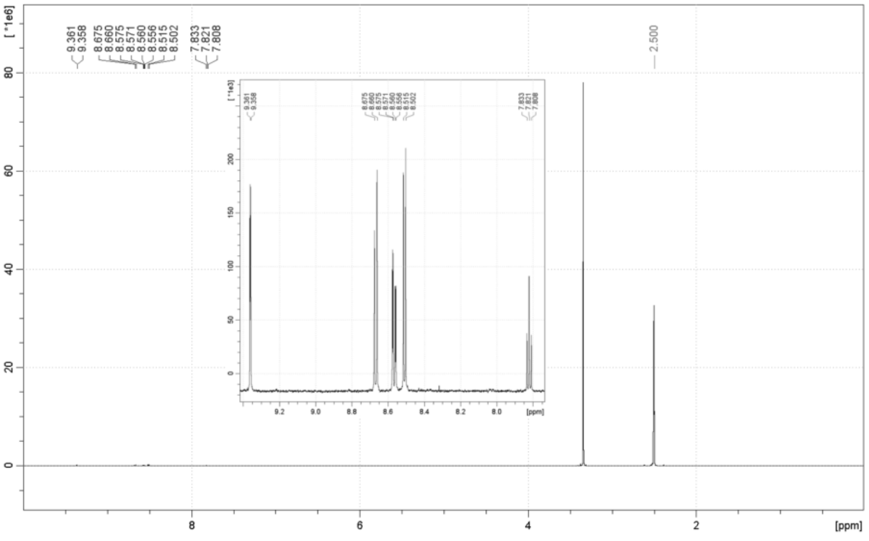

Figure S5 Proton NMR spectrum of **5**.

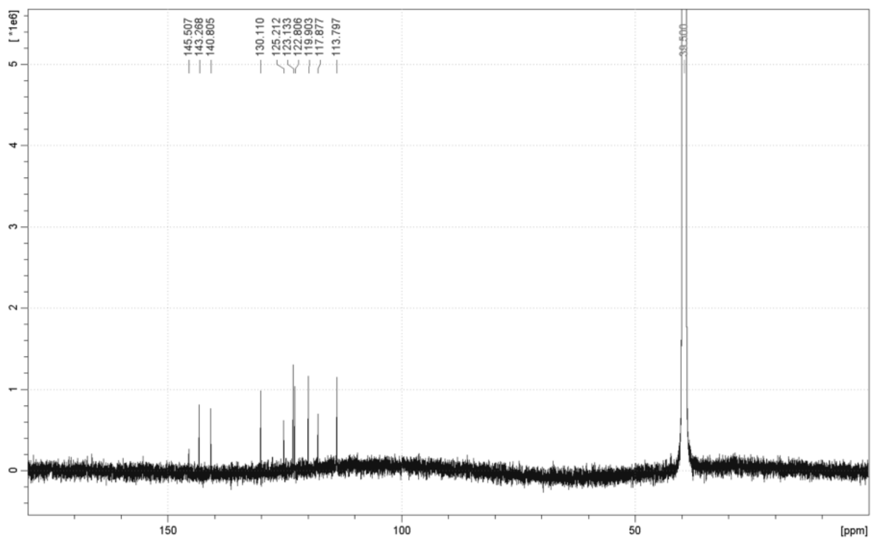

Figure S6 Carbon NMR spectrum of **5**.

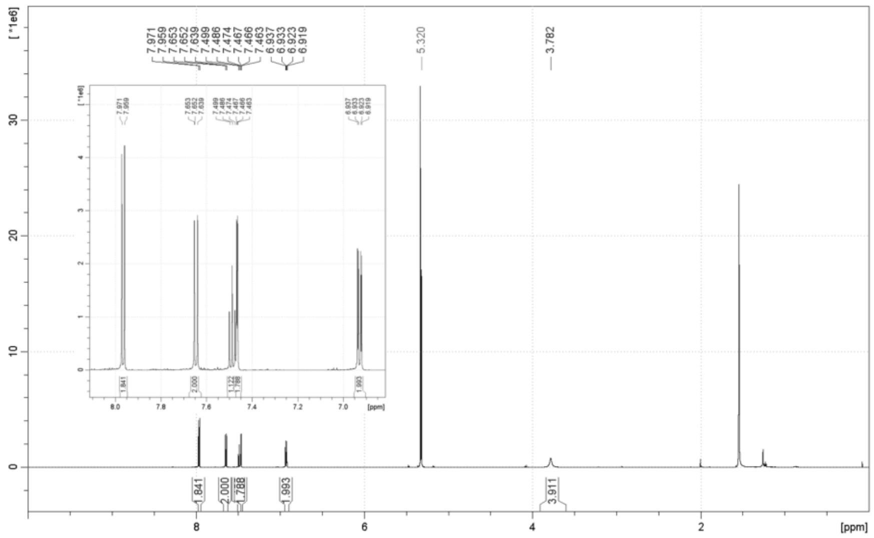

Figure S7 Proton NMR spectrum of **6**.

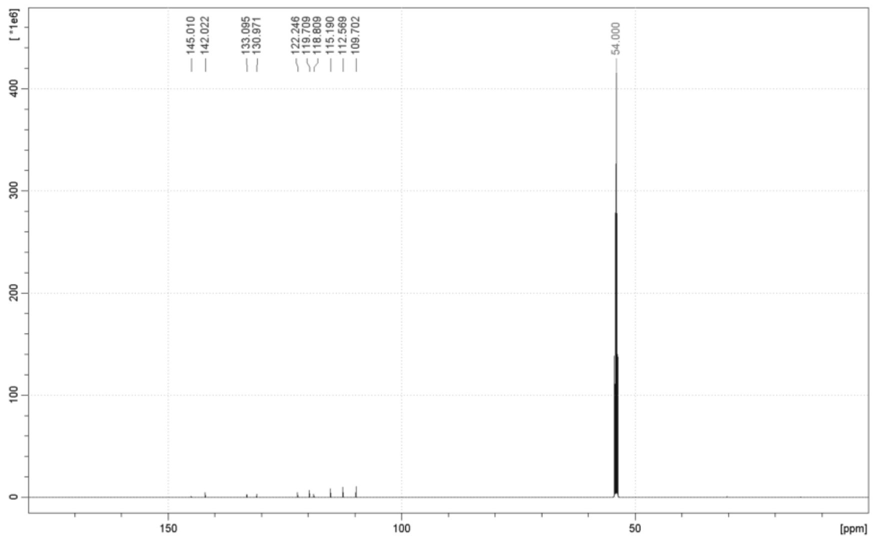

Figure S8 Carbon NMR spectrum of **6**.

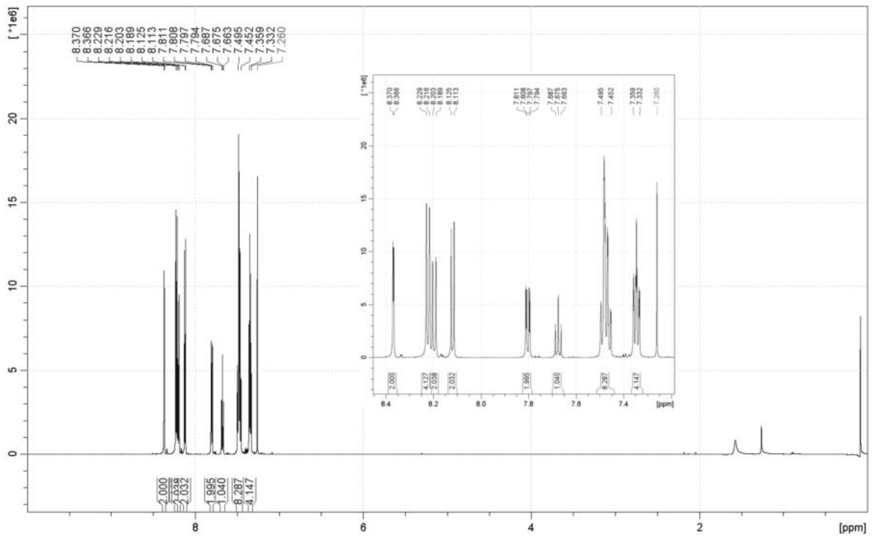

Figure S9 Proton NMR spectrum of **Cz_2ICz**.

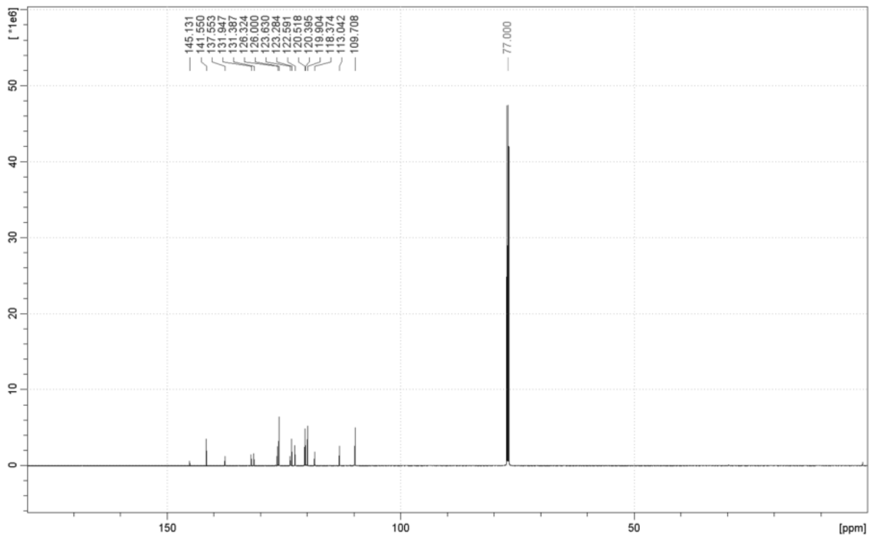

Figure S10 Carbon NMR spectrum of **Cz_2ICz**.

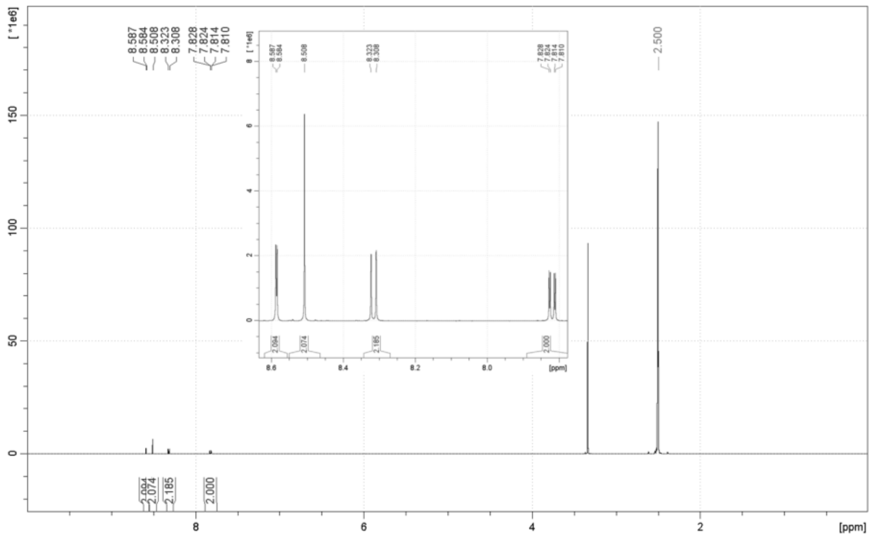

Figure S11 Proton NMR spectrum of **8**.

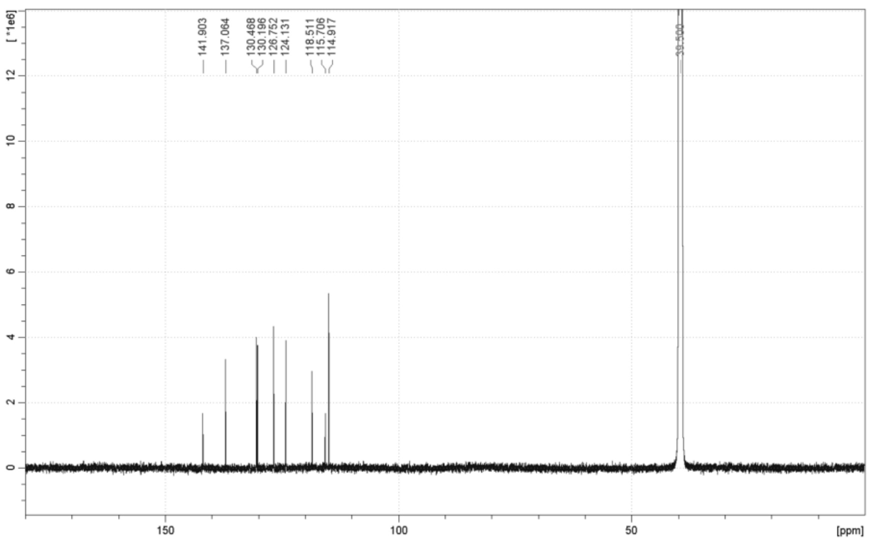

Figure S12 Carbon NMR spectrum of **8**.

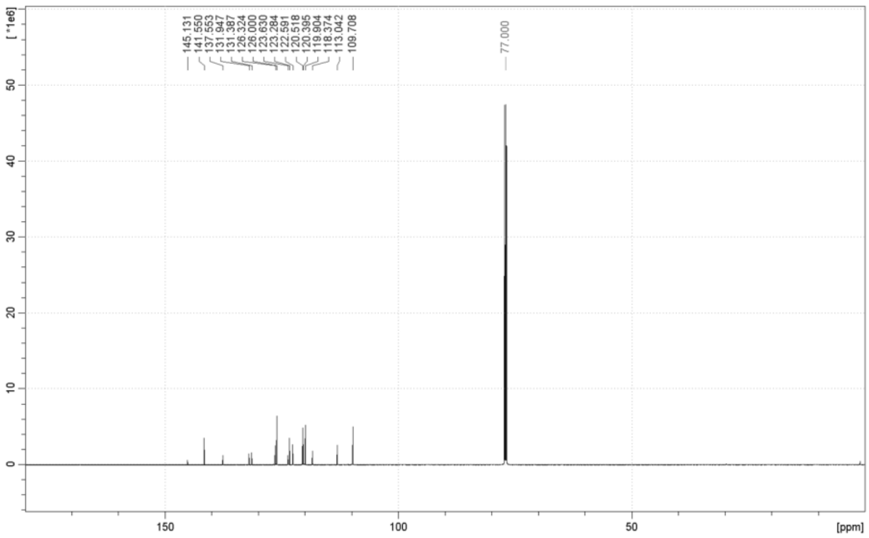

Figure S13 Proton NMR spectrum of **Cz_3ICz**.

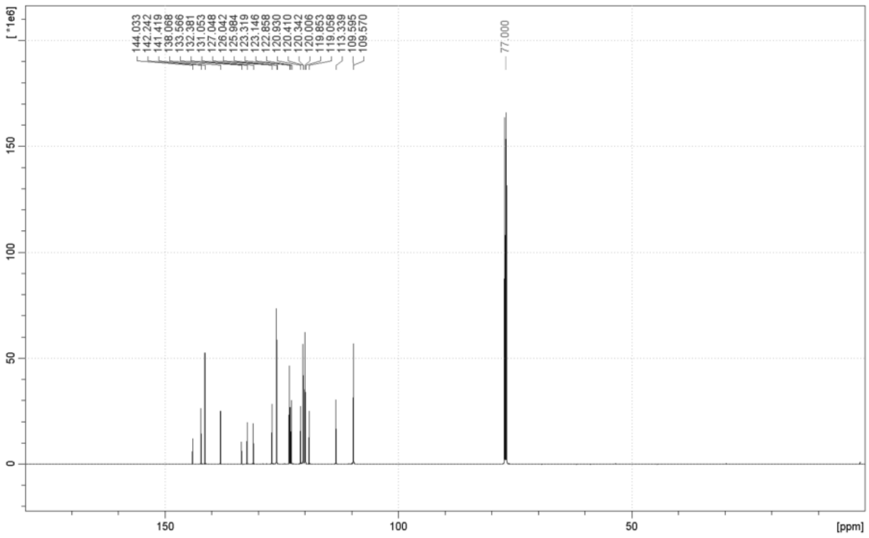

Figure S14 Carbon NMR spectrum of **Cz_3ICz**.

2. Theoretical calculations

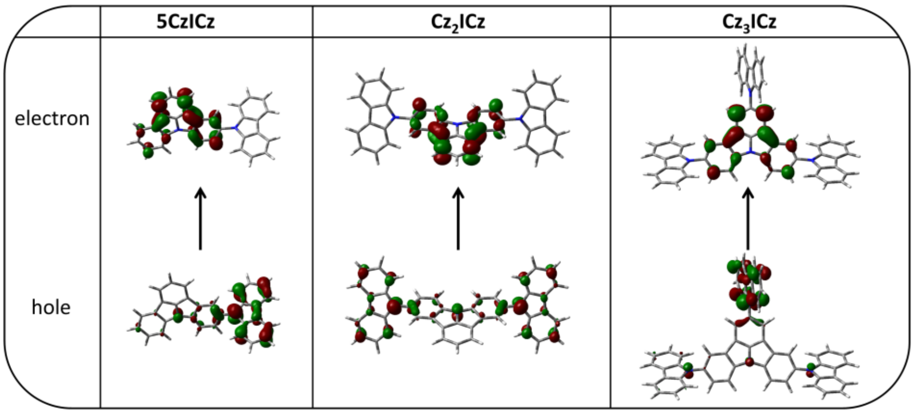

Figure S15 Natural transition orbitals (NTOs) of the $S_0 \rightarrow S_1$ transition calculated by TD-DFT at the optimized S_0 geometry.

3. Thermal characterization

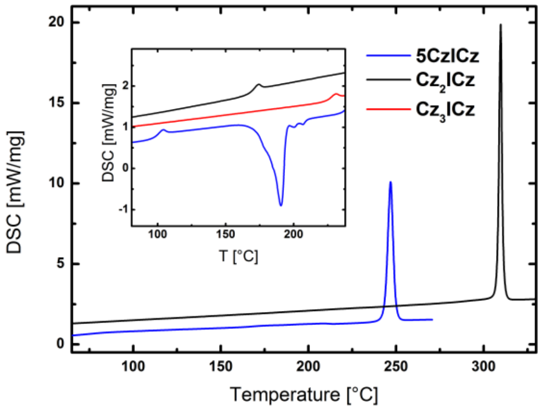

Figure S16 DSC analysis of the developed host materials (insert: second heating cycle).

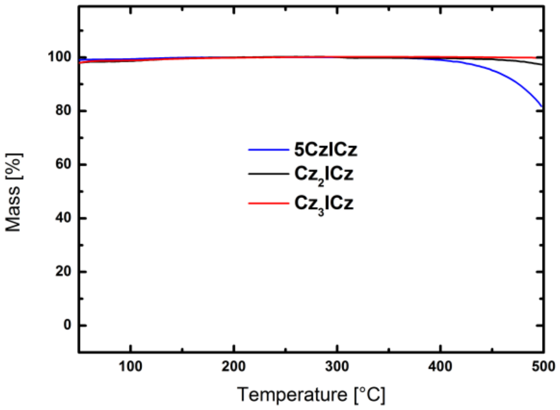

Figure S17 TGA analysis of the developed host materials.

4. Electrochemical characterization

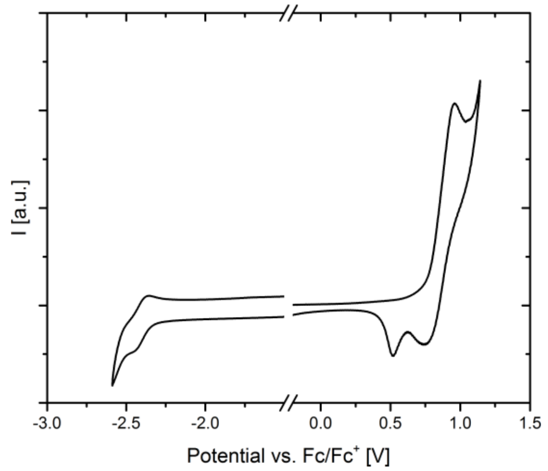

Figure S18 CV curve of **5CzICz**.

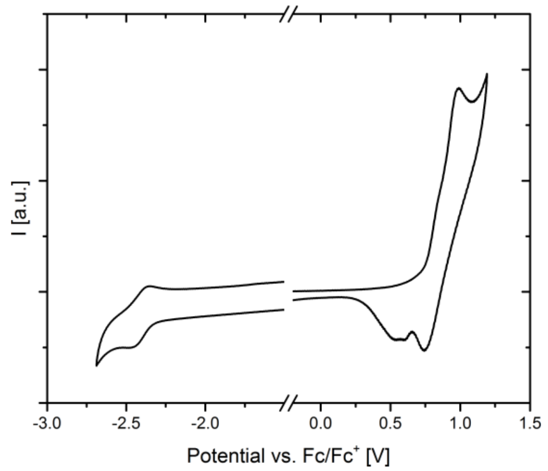

Figure S19 CV curve of **Cz_2ICz**.

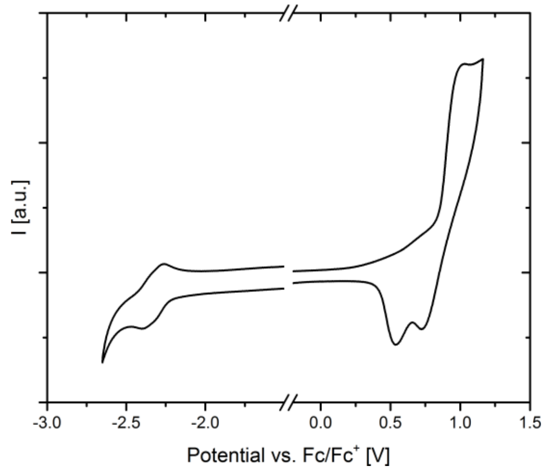

Figure S20 CV curve of **Cz_3ICz**.

5. Electroluminescent spectra

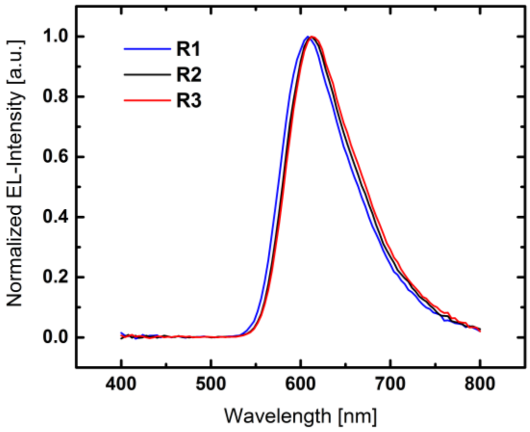

Figure S21 EL spectra of devices **R1-3** at a driving voltage of 8V.

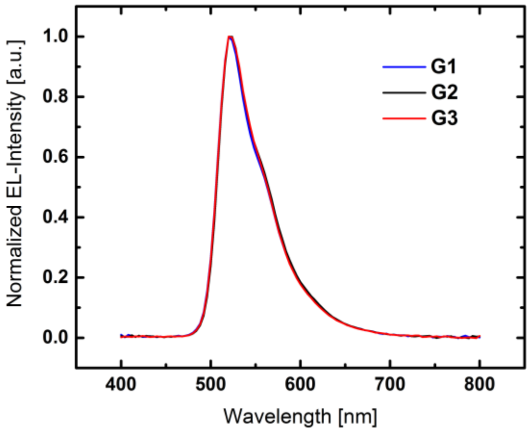

Figure S22 EL spectra of devices **G1-3** at a driving voltage of 8V.

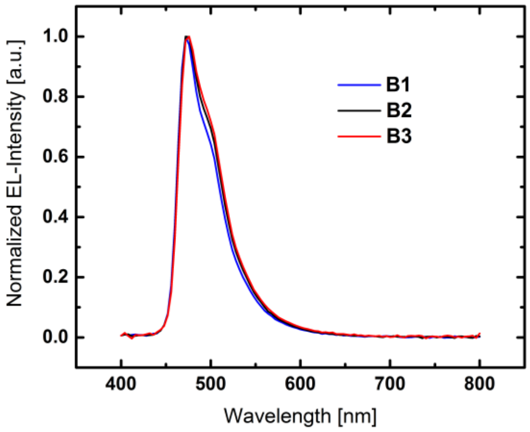

Figure S23 EL spectra of devices **B1-3** at a driving voltage of 8V.

6. Photophysical characterization

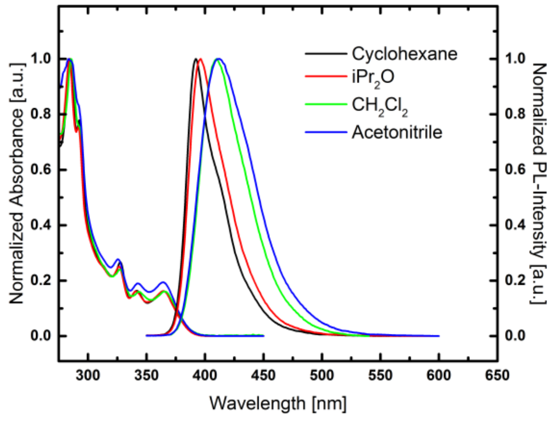

Figure S24 Normalized absorption and emission spectra of **5CzICz** in various solvents.

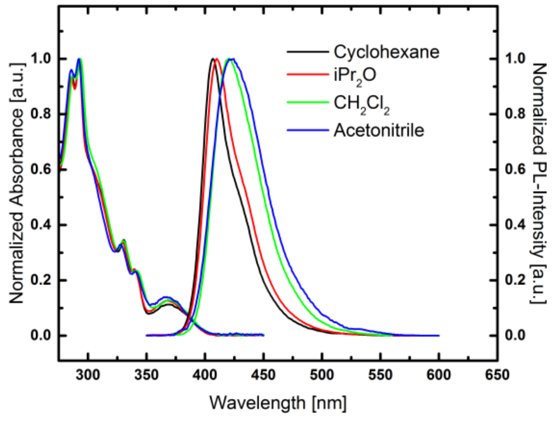

Figure S25 Normalized absorption and emission spectra of **Cz_2ICz** in various solvents.

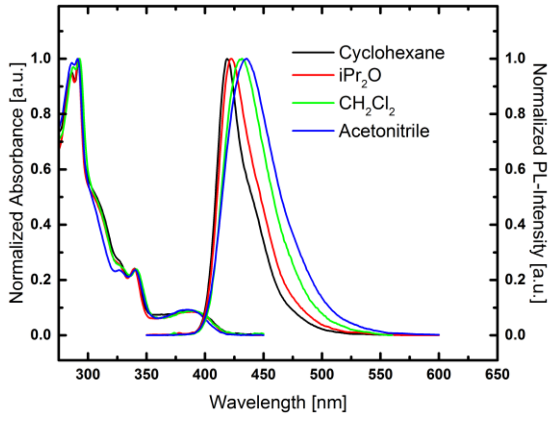

Figure S26 Normalized absorption and emission spectra of **Cz_3ICz** in various solvents.
